# Pathophysiological Mechanisms and Clinical Controversies of Sodium-Induced Hypertension: A Multi-Systemic Perspective

**DOI:** 10.3390/nu18121945

**Published:** 2026-06-16

**Authors:** Hyeong Rok Yun, Manish Kumar Singh, Sunhee Han, Jyotsna S. Ranbhise, Sung Soo Kim, Insug Kang

**Affiliations:** 1 Department of Biochemistry and Molecular Biology, School of Medicine, Kyung Hee University, Seoul 02447, Republic of Korea; foryou018@naver.com (H.R.Y.);; 2 Biomedical Science Institute, Kyung Hee University, Seoul 02447, Republic of Korea; 3 Department of Biomedical Science, Graduate School, Kyung Hee University, Seoul 02447, Republic of Korea

**Keywords:** salt sensitivity, precision nutrition, hypertension

## Abstract

Hypertension remains the primary modifiable driver of global cardiovascular morbidity, yet the long-standing paradigm of universal sodium restriction is increasingly challenged by the intricate biological heterogeneity of salt sensitivity. This review elucidates the evolving pathophysiological landscape of sodium-induced hypertension, transcending the classical Guytonian pressure-natriuresis model to incorporate emerging evidence of endothelial glycocalyx degradation, non-osmotic interstitial sodium sequestration, and gut–immune axis dysregulation. Furthermore, we critically interrogate the epidemiological “salt controversy,” examining how methodological artifacts—specifically the systematic biases inherent in spot urine sampling—may contribute to the observed J-shaped associations between sodium intake and clinical outcomes. By integrating the modulatory role of the dietary sodium-to-potassium ratio and the genetic/epigenetic determinants of individual salt-sensitive phenotypes, we propose a paradigmatic shift toward a precision nutrition approach.

## 1. Introduction

Hypertension remains the leading modifiable risk factor for global cardiovascular morbidity and mortality, driving the pathogenesis of ischemic heart disease, stroke, and chronic kidney disease [1]. Traditionally, the continuous and linear relationship between dietary sodium intake and blood pressure elevation has been regarded as a fundamental physiological postulate [2]. Major public health organizations advocate dietary sodium reduction, with recommendations commonly targeting intakes below 2.0 to 2.3 g/day of sodium (approximately 5.0 to 5.8 g/day of salt). However, this ‘one-size-fits-all’ paradigm has increasingly been questioned, as emerging evidence indicates substantial interindividual heterogeneity in sodium responsiveness and ongoing debate regarding the shape of the association between sodium intake and cardiovascular outcomes [3,4,5,6]. Furthermore, a reductionist emphasis on sodium restriction may underappreciate the interdependent roles of sodium and potassium, as well as the importance of the overall dietary pattern in shaping blood pressure and cardiovascular risk [7,8]. Unraveling these intricate interdependencies is imperative for refining evidence-based guidelines that optimize cardiovascular protection while mitigating unintended metabolic allostatic load [9,10,11]. This review synthesizes the current evidence regarding the pathophysiological mechanisms of sodium-induced hypertension. We delineate the distinct mechanisms driving salt sensitivity, critically evaluate the epidemiological controversies surrounding severe sodium restriction, and propose an integrated framework for personalized dietary prescription.

## 2. Molecular and Pathophysiological Mechanisms of Sodium-Induced Hypertension

### 2.1. The Classical Guytonian Model and Renal Hemodynamics

The traditional paradigm of sodium-induced hypertension is predicated upon the Guytonian model of renal pressure-natriuresis. Under this paradigm, the kidney is conceptualized as the principal determinant of long-term blood pressure regulation [12,13,14]. Upon surpassing the renal excretory threshold, dietary sodium intake precipitates a hyperosmotic challenge and an elevation in plasma osmolality. This hyperosmotic stimulus promotes the secretion of arginine vasopressin (AVP) and stimulates compensatory polydipsia, culminating in extracellular fluid (ECF) volume expansion [15,16]. The subsequent increase in venous return and cardiac output elicits a myogenic autoregulatory response across the peripheral vasculature, manifesting as systemic vasoconstriction. This cascade ultimately augments systemic vascular resistance, thereby sustaining arterial hypertension [12,17,18]. While this volume-centric model remains fundamentally robust, it falls short of encapsulating the full complexity of sodium-induced cardiovascular injury, particularly in salt-sensitive phenotypes manifesting hypertension in the absence of overt ECF expansion [3,19].

### 2.2. Endothelial Dysfunction and the Glycocalyx

The mechanistic landscape of sodium toxicity has expanded beyond systemic hemodynamics to encompass direct, detrimental effects on endothelial homeostasis. Elevated extracellular sodium directly antagonizes endothelial nitric oxide synthase (eNOS) activity, diminishing nitric oxide (NO) bioavailability while fueling the accrual of reactive oxygen species (ROS) [20,21,22]. Beyond enzymatic inhibition, excessive sodium mediates the attrition of the endothelial glycocalyx, a polyanionic glycosaminoglycan matrix that interfaces with the vascular lumen to maintain endothelial integrity. The endothelial glycocalyx functions as a mechanosensor and mechanotransducer of fluid shear stress and serves as a selective barrier to vascular permeability [23,24]. Elevated extracellular sodium levels neutralize the electronegative charge density of the polyanionic glycosaminoglycans, thereby inducing the structural collapse of the glycocalyx [22,25]. This disruption attenuates shear-mediated NO release, increases vascular permeability, and promotes subendothelial entry of circulating lipoproteins and inflammatory mediators, thereby contributing to arterial stiffening and endothelial dysfunction [26,27,28].

### 2.3. Tissue Sodium Storage and Immune Activation

The classical understanding of sodium homeostasis has been fundamentally expanded by recent evidence establishing the existence of nonosmotic sodium sequestration within the interstitium [29,30]. High-resolution ^23^Na magnetic resonance imaging (MRI) confirms that the skin and skeletal muscle serve as major physiological reservoirs where sodium is stored independent of water retention. This sequestration is primarily mediated by the electrostatic binding of sodium cations to highly sulfated, polyanionic glycosaminoglycans within the extracellular matrix [31,32,33]. Within the cutaneous microenvironment, localized hypertonicity triggers the activation of the tonicity-responsive enhancer binding protein (TonEBP), which serves as a master transcriptional regulator of the osmotic stress response [34,35]. Subsequent TonEBP signaling orchestrates the secretion of vascular endothelial growth factor-C (VEGF-C) by mononuclear phagocytes. This growth factor facilitates lymphatic capillary hyperplasia, which functions as a compensatory mechanism to enhance interstitial sodium clearance and maintain electrolyte balance [30]. However, when this lymphatic buffering capacity is compromised or overwhelmed, the persistent accumulation of sodium induces a phenotypic shift in macrophages toward a proinflammatory state. This immune activation serves as a critical link between tissue sodium storage and the exacerbation of salt-sensitive hypertension [36,37].

### 2.4. The Gut–Immune Axis and Microbiome Dysbiosis

Beyond systemic and vascular mechanisms, high sodium intake exerts pathological effects through the gut–immune axis [38,39]. Excessive sodium consumption induces a state of intestinal dysbiosis, primarily characterized by a significant depletion of beneficial commensal bacteria, most notably Lactobacillus species. This microbial perturbation impairs the synthesis of short-chain fatty acids (SCFAs), which serve as essential metabolites for maintaining the structural integrity of the intestinal barrier [40,41,42,43]. Consequently, increased intestinal permeability facilitates the translocation of microbial antigens into the lamina propria, which promotes the differentiation and activation of proinflammatory T helper 17 (Th17) cells. These activated lymphocytes secrete interleukin-17 (IL-17), a cytokine that enters the systemic circulation to trigger vascular inflammation and enhance renal sodium reabsorption. This mechanism identifies the gut microbiome as a pivotal intermediary that translates dietary sodium loading into systemic hypertensive stimuli [44,45]. Nevertheless, the clinical translation of the gut microbiome–immune axis in salt-sensitive hypertension remains at an early stage. Much of the mechanistic evidence linking high sodium intake to Lactobacillus depletion, Th17 activation, and IL-17–mediated vascular or renal dysfunction derives from experimental models or small mechanistic human studies. Although these studies provide biologically plausible pathways by which dietary sodium may influence systemic inflammation and blood pressure regulation, large prospective human studies and randomized clinical trials directly demonstrating that microbiome modulation improves salt-sensitive hypertension are still limited [40,46,47]. Moreover, the human gut microbiome is strongly influenced by ethnicity, habitual dietary pattern, medication exposure, age, metabolic disease, and environmental factors, making it difficult to isolate sodium-specific effects in clinical populations. Accordingly, the gut–immune axis should currently be viewed as a promising mechanistic framework rather than an established clinical target for routine hypertension management [39,48,49].

### 2.5. Central Sodium Sensing and Sympathetic Overactivity

**Figure 1:**
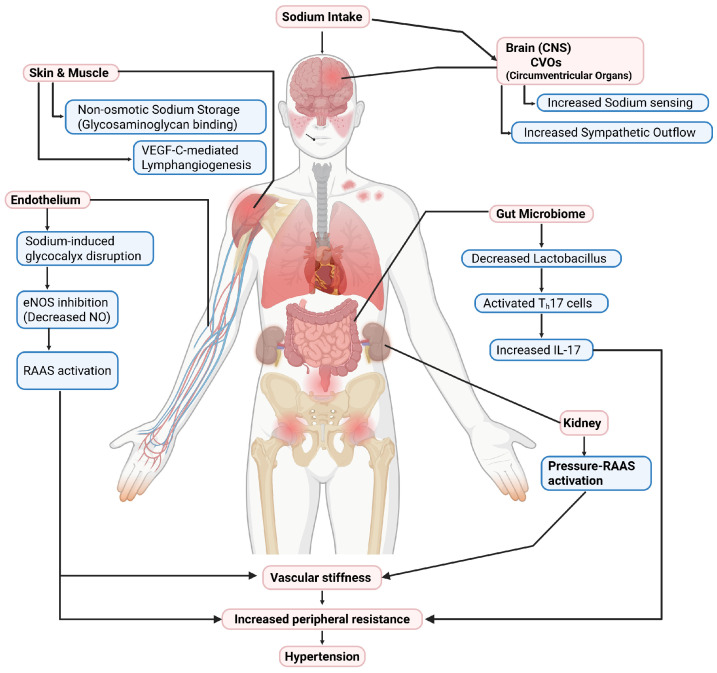
Multi-organ Pathophysiological Mechanisms of Sodium-Induced Hypertension. This schematic diagram illustrates a complex network of multi-organ interactions, moving beyond the classical renal-centric model of blood volume expansion. High sodium intake activates not only renal-pressure–natriuresis and renin–angiotensin–aldosterone system (RAAS) pathways but also systemic mechanisms in other organs. In the Brain (CNS), Circumventricular Organs (CVOs) sense increased sodium levels, leading to elevated Sympathetic Outflow. Within the Gut Microbiome, a decrease in Lactobacillus leads to activated T_h_17 cells and increased secretion of IL-17, promoting inflammation. In Skin & Muscle, sodium is stored non-osmotically through Glycosaminoglycan binding, and vascular endothelial growth factor-C (VEGF-C)-mediated lymphangiogenesis is induced. In the Endothelium, sodium causes disruption of the Glycocalyx and inhibits endothelial nitric oxide synthase (eNOS), thereby reducing Nitric Oxide (NO) production. These interacting mechanisms collectively increase vascular stiffness and total peripheral resistance, ultimately leading to hypertension. The diagram presents an integrated model that also incorporates the contribution of global systemic inflammation to this pathological process (figure created in Biorender. Hyeong Rok Yun. (2026) https://app.biorender.com/illustrations/canvas-beta/69d7c63a2021da06ecaf7bd9, accessed on 14 April 2026).

The central nervous system (CNS) functions as an integrative hub for sodium homeostasis, providing a rapid response regulatory mechanism that complements renal long-term blood pressure control [50,51]. Specialized brain regions, specifically the circumventricular organs (CVOs), lack a traditional blood–brain barrier, which permits the direct sensing of fluctuations in systemic sodium concentrations [52]. Elevated sodium levels within the cerebrospinal fluid activate the brain renin–angiotensin system and induce the synthesis of endogenous ouabain-like compounds. This signaling cascade enhances the neuronal firing rate within the hypothalamic paraventricular nucleus (PVN), resulting in a sustained elevation of sympathetic outflow [51,53,54]. This neurogenic component of salt-sensitive hypertension explains the rapid blood pressure excursions observed following sodium loading, which frequently occur independently of immediate expansions in extracellular fluid volume [37,55]. The integrated, multi-systemic pathophysiological mechanisms of sodium-induced hypertension, encompassing renal, vascular, interstitial, immune, and neural pathways, are synthesized in Figure 1.

## 3. The Salt Controversy: Linear vs. J-Shaped Relationships

### 3.1. The Traditional Linear Paradigm

The traditional linear paradigm governing sodium intake is predicated upon the evidentiary foundation of short-term dietary intervention trials, exemplified by the Dietary Approaches to Stop Hypertension (DASH) Sodium trial [56]. These investigations unequivocally establish that sodium restriction elicits a dose-dependent reduction in blood pressure across both normotensive and hypertensive cohorts. This paradigm operates on the foundational assumption that intermediate hemodynamic improvements translate linearly into a proportional decrease in long-term cardiovascular mortality and adverse clinical outcomes. Under this framework, any reduction in dietary sodium is viewed as a therapeutic benefit, as lower blood pressure levels are expected to correlate directly with a reduced risk of ischemic heart disease and stroke. Consequently, the linear model serves as the primary scientific rationale for population-wide initiatives aimed at aggressive and universal sodium restriction [2,57]. Importantly, the sodium controversy should not be interpreted as negating the clinical value of sodium reduction. Current international guidelines continue to recommend reducing excessive sodium intake as a core lifestyle strategy for hypertension prevention and management, particularly in individuals with hypertension, high cardiovascular risk, or salt-sensitive phenotypes [58,59]. The key unresolved issue is not whether excessive sodium intake should be reduced, but whether progressively lower sodium targets provide proportional long-term cardiovascular benefit across all populations. Therefore, population-level reduction in excessive sodium intake remains justified, whereas aggressive universal sodium restriction should be refined by considering baseline sodium intake, salt sensitivity, potassium intake, renal function, comorbidities, and overall dietary quality [60,61,62].

### 3.2. Epidemiological Evidence for the J-Shaped Curve

Although the blood-pressure-lowering effect of sodium reduction is well established, the relationship between habitual sodium intake and long-term cardiovascular outcomes remains debated [6]. Large observational cohorts, including the Prospective Urban Rural Epidemiology (PURE) study, have reported a J-shaped or U-shaped relationship between estimated sodium intake and cardiovascular events, suggesting excess risk at both high (5 to 6 g per day) and very low levels (below 3 g per day) of intake [63,64]. However, the interpretation of these findings remains contentious, as many such studies have relied on spot urine sampling and estimation formulas rather than repeated 24 h urine collections. When sodium exposure is assessed using more rigorous methods, particularly repeated 24 h urine sampling, the association with cardiovascular events and mortality appears more consistently positive and approximately linear [65]. Taken together, the current controversy likely reflects not only biological heterogeneity but also major differences in exposure assessment, analytical methodology, and study population characteristics.

### 3.3. Mechanistic Basis for the J-Shaped Relationship

Several biological mechanisms have been invoked to explain the adverse outcomes reported at very low levels of sodium intake. Severe sodium restriction may transiently activate counter-regulatory pathways, including the renin–angiotensin–aldosterone system (RAAS) and the sympathetic nervous system (SNS), while also influencing insulin sensitivity and lipid metabolism [66,67,68]. However, these responses alone are insufficient to account for the excess risk described in some observational studies. A growing body of evidence indicates that the apparent J-shaped relationship is, at least in part, attributable to methodological artifact, particularly the biased estimation of sodium intake from spot urine samples, as well as reverse causality in individuals with chronic illness who consume less food and sodium despite carrying a higher baseline risk of mortality [69,70]. Accordingly, the lower end of the sodium–outcome curve warrants cautious interpretation. Rather than supporting a definitive harmful threshold for sodium reduction, the available evidence underscores the need for rigorously designed prospective studies incorporating repeated 24 h urine collections to distinguish true biological effects from bias.

## 4. Inter-Individual Heterogeneity: The Concept of Salt Sensitivity

### 4.1. Definition and Genetic Determinants of Salt Sensitivity

A significant limitation of universal sodium guidelines is the inherent assumption of physiological homogeneity. In clinical reality, the blood pressure response to dietary sodium perturbations follows a Gaussian distribution, demarcating the discrete clinical phenotypes of salt sensitivity and salt resistance. Salt sensitivity of blood pressure (SSBP) is defined as a significant hemodynamic shift in response to variations in sodium intake, manifesting in approximately 50 percent of hypertensive individuals and 25 percent of the normotensive population [3,71,72]. SSBP is recognized as a complex polygenic trait influenced by diverse molecular mechanisms. Genetic polymorphisms within renal sodium transporters, specifically the epithelial sodium channel (ENaC) and the thiazide-sensitive NaCl cotransporter (NCC), serve as primary determinants of individual susceptibility [55,73]. Furthermore, variants in regulatory signaling pathways, including With No Lysine (WNK) kinases and G protein coupled receptor kinase 4 (GRK4), enhance the renal sodium reabsorption through dysregulation of distal nephron transport pathways. These alterations collectively increase susceptibility to salt-sensitive hypertension by impairing the ability of the kidney to effectively excrete a sodium load, thereby shifting pressure-natriuresis toward a requirement for higher arterial pressures to maintain sodium balance [74,75,76].

### 4.2. Influence of Aging and Metabolic Status on Salt Sensitivity

Beyond genetic predisposition, salt sensitivity of blood pressure (SSBP) is profoundly modulated by age, renal function, and metabolic status. Physiological senescence is characterized by progressive nephron loss and arterial stiffening, both of which impair the renal-pressure–natriuresis mechanism and exacerbate salt sensitivity. This age-related decline in pressure–natriuresis and renal sodium excretory capacity shifts sodium balance toward a requirement for higher arterial pressures, thereby contributing to the increased prevalence of salt sensitivity in older adults [77,78]. Furthermore, metabolic syndrome and insulin resistance are inextricably linked to the pathogenesis of SSBP. Hyperinsulinemia serves as a potent stimulus for sodium reabsorption within the proximal tubule and distal nephron through the activation of ENaC and the thiazide-sensitive NCC [74,79,80]. Simultaneously, insulin resistance is frequently accompanied by sympathetic overactivity, which further promotes renal sodium retention and increase vascular tone. Consequently, the clinical response to aggressive sodium restriction is heterogeneous and likely depends on the underlying salt-sensitive phenotype and metabolic status [51,81]. While such interventions yield substantial hemodynamic benefits in salt-sensitive cohorts, including the elderly and patients with chronic kidney disease or metabolic syndrome, they may offer negligible or potentially deleterious effects in young, healthy, salt-resistant populations [78]. This underscores the necessity of a personalized approach to nutritional therapy rather than a universal mandate.

### 4.3. Epigenetic Programming and Early-Life Origins

Individual susceptibility to salt-sensitive hypertension is frequently established during critical developmental windows through a process known as fetal programming [82,83,84]. According to the Developmental Origins of Health and Disease (DOHaD) hypothesis, excessive maternal sodium intake during gestation induces epigenetic modifications within the fetal genome, including altered DNA methylation and histone modification, thereby predisposing offspring to later hypertension [84,85,86]. These epigenetic imprints exert permanent alterations on the transcriptional regulation of renal sodium transporters, such as ENaC, and may concurrently impair the development of the nephron endowment [87,88,89]. Consequently, early-life programming may sensitize offspring to prohypertensive stimuli, resulting in an exaggerated blood pressure response to dietary sodium in later life [90,91]. This developmental trajectory suggests that salt sensitivity should not be conceptualized as a static adult trait but rather as a progressive condition viewed through a life-course perspective [84,91]. Recognizing the early-life origins of salt-sensitive cardiovascular risk is essential for identifying high-risk populations and guiding primordial prevention strategies aimed at reducing the long-term risk of cardiovascular disease [92,93]. Despite the strong biological plausibility of these genetic and epigenetic mechanisms, their current clinical utility remains limited. Variants in ENaC, NCC, WNK kinases, GRK4 and RAAS-related pathways have been associated with altered renal sodium handling and salt-sensitive blood pressure responses in selected cohorts, but most markers have not yet achieved sufficient reproducibility, effect size, or predictive accuracy to guide routine dietary sodium prescription in clinical practice. Similarly, epigenetic signatures linked to fetal programming, renal sodium transporter regulation, or neurohormonal activation are mechanistically informative but remain largely investigational. Their interpretation is further complicated by tissue specificity, age-dependent remodeling, environmental interactions, and limited availability of validated assays in accessible clinical samples. Therefore, genetic and epigenetic determinants should currently be regarded as research tools that help explain interindividual heterogeneity in salt sensitivity, rather than as fully validated biomarkers for routine precision nutrition [82,94,95]. Future studies integrating genomics, epigenomics, urinary electrolyte phenotyping, ambulatory blood pressure monitoring, and clinical outcomes will be required before these markers can be translated into actionable clinical algorithms.

### 4.4. Circadian Rhythmicity and the Non-Dipper Phenotype

The clinical significance of salt sensitivity is intrinsically linked to the circadian regulation of arterial pressure [96]. In salt-sensitive individuals, the renal capacity to excrete the daily sodium load is frequently attenuated during the diurnal phase. To achieve cumulative sodium balance, the body recruits a compensatory elevation in nocturnal blood pressure to drive pressure–natriuresis throughout the sleep period [97,98]. This hemodynamic adaptation manifests as the non-dipper phenotype, in which the normal 10 to 20 percent nocturnal decline in blood pressure is attenuated [99]. This sustained nocturnal hypertension serves as a robust independent predictor of target-organ injury, including left ventricular hypertrophy and progressive renal dysfunction [100]. Consequently, the loss of normal circadian dipping represents a key pathophysiological link between impaired sodium handling and the increased cardiovascular risk.

## 5. Translational Aspects: Beyond Isolated Sodium Restriction

### 5.1. The Modulatory Role of Dietary Potassium and the Sodium-to-Potassium Ratio

The physiological impact of sodium is profoundly modulated by the concurrent intake of potassium, as an exclusive focus on isolated sodium consumption fails to account for the broader dietary composition [60]. Extensive clinical evidence suggests that the dietary Na^+^/K^+^ ratio is more closely associated with arterial pressure and cardiovascular risk than sodium intake considered in isolation [101]. Potassium facilitates potent antihypertensive effects through several distinct and synergistic mechanisms [102]. Specifically, potassium promotes natriuresis by inhibiting the thiazide-sensitive NCC in the distal convoluted tubule, thereby enhancing urinary sodium excretion [103]. Furthermore, potassium contributes to blood pressure reduction by promoting vasodilation through hyperpolarization-dependent vascular signaling and by modulating aldosterone-dependent renal sodium handling, rather than by directly suppressing RAAS as a whole [104]. Diets rich in potassium can mitigate the adverse hemodynamic and vascular consequences of sodium loading [105]. This synergistic interaction underscores the importance of considering sodium and potassium jointly within the broader dietary matrix. Accordingly, the Na^+^/K^+^ ratio should be considered a practical and clinically informative target for dietary interventions aimed at mitigating salt-sensitive hypertension [60,101]. Although the optimal dietary Na^+^/K^+^ ratio remains incompletely defined, a molar ratio approaching 1:1 has been proposed as a potentially favorable target for cardiometabolic health. This concept is broadly consistent with public health recommendations that encourage sodium intake below approximately 2 g/day while promoting potassium intake above approximately 3.5 g/day, which corresponds to a near-equimolar sodium-to-potassium balance [60]. However, it should be emphasized that direct clinical evidence testing an aggressively targeted 1:1 Na^+^/K^+^ ratio remains limited, and most interventional trials have evaluated more pragmatic dietary approaches, such as sodium reduction, potassium-rich dietary patterns, or partial replacement of sodium chloride with potassium chloride. Therefore, the 1:1 Na^+^/K^+^ ratio should be interpreted as a conceptual and physiological benchmark rather than a universally validated therapeutic threshold [61,62,106].

### 5.2. Precision Nutrition Interventions

**Figure 2:**
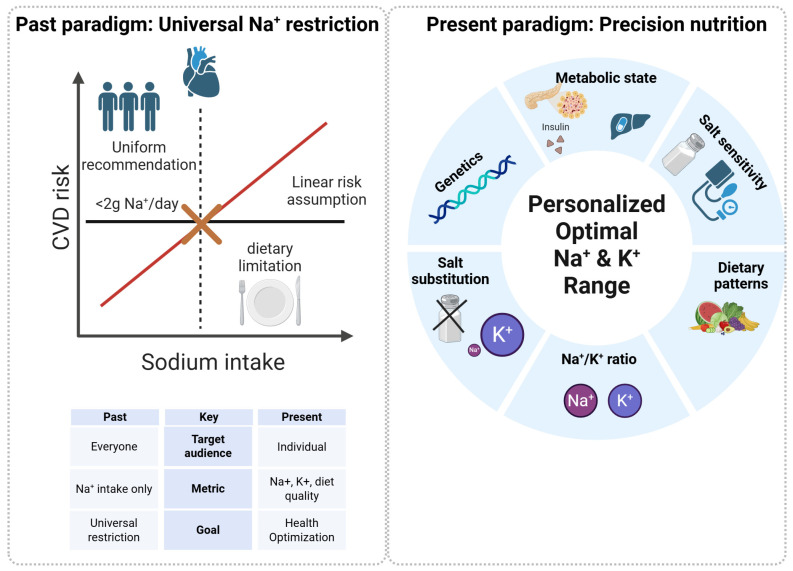
The clinical paradigm shift from universal sodium restriction to precision nutrition in hypertension management. This infographic contrasts the fundamental evolution in clinical approaches to dietary sodium management for the prevention of hypertension and cardiovascular disease (CVD). The left panel illustrates the historical “one-size-fits-all” paradigm, which relies on a simplified linear risk assumption suggesting that CVD risk increases proportionally with sodium intake for all individuals. Consequently, this model promotes a uniform recommendation of strict sodium restriction to less than 2 g per day across the entire population. In contrast, the right panel depicts the contemporary precision nutrition framework, which prioritizes a tailored strategy to identify a personalized optimal Na^+^ and K^+^ range. This multifaceted approach integrates individual genetic profiles, such as polymorphisms in the RAAS pathway, and current metabolic states, including insulin resistance and hormonal balance. Furthermore, it incorporates phenotypic assessments of salt sensitivity and emphasizes holistic dietary patterns, such as the DASH and Mediterranean diets, rather than focusing solely on nutrient isolation. Central to this paradigm is the optimization of the Na^+^/K^+^ ratio through both sodium reduction and increased potassium intake, often facilitated by salt substitution strategies using potassium-enriched alternatives. As summarized in the comparison table, this shift represents a move from universal restriction toward health optimization by treating the individual as the primary unit of clinical intervention (figure created in Biorender. Hyeong Rok Yun. (2026) https://app.biorender.com/illustrations/canvas-beta/690b51026e6e5df0b20b387c, accessed on 14 April 2026).

Interindividual variability in blood pressure responses underscores the limitations of one-size-fits-all dietary recommendations and supports a transition toward precision nutrition strategies [107,108]. Dietary patterns such as the DASH and Mediterranean diets, which are rich in potassium, calcium, and fiber, can help shift the dietary Na^+^/K^+^ ratio in a more favorable direction than that typically seen in modern Western diets [9,109,110]. The paradigmatic shift from a universal sodium restriction model toward this individualized precision nutrition approach, which integrates genetic, metabolic, and lifestyle factors, is illustrated in Figure 2. Whole-food-centered nutritional strategies may help reduce ultra-processed food intake and thereby lower exposure to the excess sodium that is commonly delivered through processed, packaged, and prepared foods [9,111]. By emphasizing overall dietary quality rather than isolated nutrient restriction, these interventions can promote a more favorable electrolyte balance without relying on excessively stringent sodium restriction [6,60]. This holistic approach ensures that sodium reduction occurs within a nutrient-dense matrix, maximizing cardiovascular protection while maintaining long-term sustainability and patient adherence.

### 5.3. Large-Scale Clinical Evidence: The Role of Salt Substitutes

The translational potential of dietary sodium–potassium modification has been reinforced by recent landmark clinical trials, most notably the Salt Substitute and Stroke Study (SSaSS). Unlike traditional strategies focused on sodium restriction, SSaSS tested a pragmatic household-level intervention in which regular salt was replaced with a potassium-enriched salt substitute composed of 75% NaCl and 25% KCl. The trial was conducted in a high-risk population in rural China, including individuals with a history of stroke or older adults with poorly controlled blood pressure. This design is clinically relevant because it evaluated a feasible real-world intervention rather than an intensive dietary counseling program [61]. SSaSS demonstrated that potassium-enriched salt substitution significantly reduced the risks of stroke, major cardiovascular events, and all-cause mortality [101]. These findings provide high-level clinical evidence that simultaneous sodium reduction and potassium augmentation can translate into cardiovascular protection beyond short-term blood pressure lowering. Mechanistically, this dual-action strategy may improve sodium-to-potassium balance, enhance natriuresis, and attenuate sodium-mediated vascular and renal injury [61,112]. This paradigm shift from negative nutrient targeting toward positive substitution may provide a pragmatic and sustainable strategy for reducing the global burden of hypertension and its associated cardiovascular complications. By prioritizing restoration of a more physiological electrolyte balance within the overall dietary matrix, this approach addresses key mechanisms underlying salt sensitivity while supporting longer-term adherence across broad populations [6,113]. However, the generalizability and safety of potassium-enriched salt substitution should be interpreted carefully. The SSaSS population had high baseline cardiovascular risk and was drawn from rural communities where discretionary salt use during home cooking represents a major source of sodium intake. Therefore, the magnitude of benefit may differ in populations with lower baseline sodium intake or in countries where most sodium exposure comes from processed and restaurant foods rather than household salt [114,115]. In addition, potassium-enriched salt substitutes should not be considered universally risk-free. In patients with salt-sensitive hypertension, coexisting chronic kidney disease and concomitant use of medications that impair renal potassium excretion, including RAAS inhibitors, mineralocorticoid receptor antagonists, and potassium-sparing diuretics, may increase susceptibility to hyperkalemia during indiscriminate potassium supplementation. Therefore, the clinical implementation of potassium-enriched salt substitutes should be individualized on the basis of renal function, baseline serum potassium concentration, concomitant medications, and overall hyperkalemia risk [116,117].

## 6. Methodological Considerations and Future Directions

The fundamental challenge in resolving the sodium controversy lies in the methodological limitations inherent in assessing long-term dietary sodium intake. While the collection of multiple nonconsecutive 24 h urine samples remains the established gold standard for quantifying sodium consumption, logistical and financial constraints often necessitate the use of fasting morning spot urine samples in large-scale epidemiological investigations. These studies frequently rely on predictive equations, such as the Kawasaki or Tanaka formulas, to estimate 24 h sodium excretion from spot urine samples [69,118,119]. However, these predictive equations exhibit systematic proportional bias, generally overestimating 24 h sodium excretion at lower intake levels while underestimating it at higher levels [120]. This systematic error may artificially generate or exaggerate an apparent J-shaped association between sodium intake and mortality. Furthermore, observational associations may be confounded by reverse causality, whereby individuals with preexisting chronic illness and intrinsically higher mortality risk may consume less energy and sodium as a consequence of their underlying disease [70,121]. To definitively characterize the optimal therapeutic range for sodium intake, future prospective randomized controlled trials incorporating rigorous multiday 24 h urine collections will be essential [119]. Moreover, the identification of feasible clinical biomarkers for stratifying salt-sensitive phenotypes remains a key prerequisite for translating precision nutrition interventions into clinical practice. Shifting the focus from universal population mandates toward targeted strategies for salt-sensitive individuals will likely provide the most efficacious approach for mitigating the global impact of hypertensive cardiovascular disease [71,122].

## 7. Conclusions

The transition from universal sodium mandates toward precision nutrition represents a critical paradigm shift that prioritizes individual salt sensitivity and the dietary sodium-to-potassium ratio over absolute restriction. By integrating these personalized strategies with a holistic focus on whole-food matrices, clinicians can optimize cardiovascular protection while mitigating the systemic metabolic stressors associated with extreme sodium depletion.

## Data Availability

No new data were created or analyzed in this study.

## Abbreviations

The following abbreviations are used in this manuscript:

AVP	Arginine vasopressin
CNS	Central nervous system
CVOs	Circumventricular organs
DASH	Dietary Approaches to Stop Hypertension
DOHaD	Developmental Origins of Health and Disease
ECF	Extracellular fluid
ENaC	Epithelial sodium channel
eNOS	Endothelial nitric oxide synthase
GRK4	G protein-coupled receptor kinase 4
IL-17	Interleukin-17
MRI	Magnetic resonance imaging
NCC	Thiazide-sensitive NaCl cotransporter
NO	Nitric oxide
PURE	Prospective Urban Rural Epidemiology
PVN	Paraventricular nucleus
RAAS	Renin–angiotensin–aldosterone system
ROS	Reactive oxygen species
SCFAs	Short-chain fatty acids
SNS	Sympathetic nervous system
SSaSS	Salt Substitute and Stroke Study
SSBP	Salt sensitivity of blood pressure
Th17	T helper 17
TonEBP	Tonicity-responsive enhancer-binding protein
VEGF-C	Vascular endothelial growth factor-C
WNK	With No Lysine (kinase)
